# Association of mortality with fludrocortisone addition to hydrocortisone treatment among septic shock patients: a propensity score matching analysis

**DOI:** 10.3389/fmed.2023.1190758

**Published:** 2023-05-09

**Authors:** Xiaoxiao Cheng, Zhiqin Fu, Yiting Liu, Xiaoyu Zheng, Tianyang Hu

**Affiliations:** ^1^Department of Ultrasound, The Second Affiliated Hospital of Chongqing Medical University, Chongqing Key Laboratory of Ultrasound Molecular Imaging, Institute of Ultrasound Imaging, Chongqing, China; ^2^Department of Orthopedic Surgery, Anyue Hospital of Traditional Chinese Medicine, Second Ziyang Hospital of Traditional Chinese Medicine, Ziyang, China; ^3^Department of Critical Care Medicine, The Second Affiliated Hospital of Chongqing Medical University, Chongqing, China; ^4^School of Clinical Medicine, Chongqing Medical and Pharmaceutical College, Chongqing, China; ^5^Precision Medicine Center, The Second Affiliated Hospital of Chongqing Medical University, Chongqing, China

**Keywords:** septic shock, corticosteroid, hydrocortisone, fludrocortisone, mortality

## Abstract

**Background:**

Several clinical trials of corticosteroids have been carried out in the treatment of septic shock, however, the therapeutic effect of the most widely used hydrocortisone is still controversial, and no studies have directly compared hydrocortisone versus hydrocortisone plus fludrocortisone for patients with septic shock.

**Methods:**

Baseline characteristics and treatment regimens of patients with septic shock treated with hydrocortisone from the Medical Information Mart for Intensive Care-IV database were collected. Patients were divided into hydrocortisone treatment groups and hydrocortisone plus fludrocortisone treatment groups. The primary outcome was 90-day mortality, and secondary outcomes included 28-day mortality, in-hospital mortality, length of hospital stay, and length of intensive care unit (ICU) stay. Binomial Logistic regression analysis was performed to identify independent risk factors for mortality. Survival analysis was performed and Kaplan–Meier curves were drawn for patients in different treatment groups. Propensity score matching (PSM) analysis was performed to reduce bias.

**Results:**

Six hundred and fifty three patients were enrolled, of which 583 were treated with hydrocortisone alone, and 70 with hydrocortisone plus fludrocortisone. After PSM, 70 patients were included in each group. The proportion of patients with acute kidney injury (AKI) and the proportion of renal replacement therapy (RRT) treatment in the hydrocortisone plus fludrocortisone group were higher than those in the hydrocortisone alone group, and there was no significant difference in other baseline characteristics. Compared with hydrocortisone alone, hydrocortisone plus fludrocortisone did not reduce the 90-day mortality (after PSM, relative risk/RR = 1.07, 95%CI 0.75–1.51), 28-day mortality (after PSM, RR = 0.82, 95%CI 0.59–1.14) and in-hospital mortality (after PSM, RR = 0.79, 95%CI 0.57–1.11) of the enrolled patients, nor did it affect the length of hospital stay (after PSM, 13.9 days vs. 10.9 days, *p* = 0.34) and ICU stay (after PSM, 6.0 days vs. 3.7 days, *p* = 0.14), and the survival analysis showed no statistically significant difference in the corresponding survival time. After PSM, binomial Logistic regression analysis showed that SAPS II score was an independent risk factor for 28-day morality (OR = 1.04, 95%CI 1.02–1.06, *p* < 0.01) and in-hospital morality (OR = 1.04, 95%CI 1.01–1.06, *p* < 0.01), while hydrocortisone plus fludrocortisone was not an independent risk factor for 90-day mortality (OR = 0.88, 95%CI 0.43–1.79, *p* = 0.72), 28-day morality (OR = 1.50, 95%CI 0.77–2.91, *p* = 0.24), or in-hospital morality (OR = 1.58, 95%CI 0.81–3.09, *p* = 0.18).

**Conclusion:**

In the treatment of patients with septic shock, hydrocortisone plus fludrocortisone did not reduce 90-day mortality, 28-day mortality, and in-hospital mortality compared with hydrocortisone alone, and had no effect on the length of hospital stay and ICU stay.

## 1. Background

Sepsis is defined as life-threatening organ dysfunction resulting from a dysregulated host response to infection, while septic shock is an important subset of sepsis with particularly severe circulatory, cellular, and metabolic disturbances. The short-term mortality of septic shock is 45–50% (1, 2). Early hemodynamic and respiratory resuscitation and appropriate anti-infective therapy are the main treatment options for septic shock (3). Sepsis is associated with a dysregulated response on the hypothalamic–pituitary–adrenal axis (4), thus corticosteroids have been used to treat patients with severe infections, especially in patients with septic shock (with hydrocortisone being the most widely used). However, the survival benefit of patients is highly controversial. Previous studies have reported that only about one-third of physicians believe that corticosteroids can improve survival in patients with septic shock (5).

Several landmark studies had investigated the impact of hydrocortisone on outcomes in patients with septic shock. In 2002, a study by Annane et al. (6) reported the use of low-dose hydrocortisone (50-mg intravenous bolus every 6 h) plus fludrocortisone (50-micro g tablet once daily) in patients with septic shock. In the 7-day treatment period, this therapy was found to significantly reduce the 28-day risk of death in patients with septic shock and relative adrenal insufficiency compared with placebo, without increasing adverse events. In 2008, Sprung et al. (7) investigated hydrocortisone (50-mg intravenous bolus every 6 h, tapered on day 6) versus placebo in the treatment of patients with septic shock (the Corticosteroid Therapy of Septic Shock trial, abbreviated as the CORTICUS trial), and found that although hydrocortisone accelerated shock reversal, it did not improve 28-day survival. And in 2018, Annane et al. (8) reported that 90-day all-cause mortality was lower in septic patients treated with hydrocortisone (50-mg intravenous bolus every 6 h) plus fludrocortisone (50-micro g tablet once daily) for 7 days compared with placebo (the Activated Protein C and Corticosteroids for Human Septic Shock trial, abbreviated as the APROCCHSS trial). Overall, the survival benefit of hydrocortisone-treated septic shock patients in the above studies is controversial, and hydrocortisone with or without fludrocortisone may be a key factor affecting the survival benefit of the patients. However, no studies to date have directly compared the survival benefit of hydrocortisone versus hydrocortisone plus fludrocortisone in patients with septic shock. This study attempts to address this question based on a large public clinical database.

## 2. Methods

### 2.1. Database

This study is based on the Medical Information Mart for Intensive Care (MIMIC), a database comprising deidentified health-related data from patients who were admitted to the critical care units of the Beth Israel Deaconess Medical Center. The MIMIC database was updated to MIMIC-IV 2.0 version on June 13, 2022^1^. The database has collected more than 70,000 hospitalization information of critically ill patients from 2008 to 2019, and is currently the largest critical medical database. Since its data collection and input processes were all carried out by professionally trained personnel, its data quality is extremely high.

This study complies with the provisions of the Declaration of Helsinki. The author, Tianyang Hu, has passed the “Protecting Human Research Participants” exam (certification No. 37474354) on the website of the National Institutes of Health (NIH), signed a data use agreement, and the study has been approved by an affiliate of the Massachusetts Institute of Technology (No. 27653720). All patient identification information (including name, home address, etc.) in the database is de-identified, so there is no need to obtain the informed consent of the patients.

### 2.2. Study population and data extraction

Navicat Premium software (version 15.0) were used to extract the basic characteristics of the included patients from MIMIC-IV database by SQL (Structure query language). The inclusion criteria for this study were as follows: septic shock was diagnosed after admission, hemodynamically unstable after adequate fluid resuscitation and vasoactive drug therapy, and corticosteroid therapy was required after physician evaluation, and the corticosteroid drug was hydrocortisone (alone or plus fludrocortisone). Disease codes for septic shock include 78,552 (septic shock, international classification of diseases/ICD 9), R6521 (severe sepsis with septic shock, ICD 10), and T8112XA (postprocedural septic shock, initial encounter, ICD 10). The exclusion criteria were as follows: non-first-time admissions, non-first-time ICU admissions; non-adult patients; patients with a hospital stay of less than 1 day; patients treated with corticosteroids for other reasons.

We extracted the following data of the patients: age, gender, race, admission time, discharge time, intensive care unit (ICU) admission time, ICU discharge time, Charlson comorbidity index (CCI), acute kidney injury or not, Sequential Organ Failure Assessment (SOFA) score in the first 24 h of admission, Simplified Acute Physiology Score II (SAPS II) score and Glasgow Coma Scale (GCS) score, other treatments in addition to corticosteroids: vasopressors (epinephrine, norepinephrine, dopamine, dobutamine), whether mechanical ventilation was used, whether extracorporeal membrane oxygenation (ECMO) was used, and whether renal replacement therapy (RRT) was used. Details of systemic treatment by hydrocortisone/fludrocortisone for patients with septic shock were extracted, include the average daily dose of the drug and the treatment duration. Hydrocortisone and fludrocortisone need to be administered on the same day with an interval of no more than 12 h, otherwise they cannot be considered as a combination. Python software (version 3.9) was used for processing the above data, and manual errata and cross-checking were performed by two researchers. The treatment duration (day) of systemic corticosteroid was defined as “end date minus start date plus 1.” If the “start date” and the “end date” were the same, the treatment duration was equal to 1 day. Data with unknown route of corticosteroids were deleted.

### 2.3. Outcomes

Patients were divided into hydrocortisone treatment groups and hydrocortisone plus fludrocortisone treatment groups. The primary outcome was 90-day mortality, and secondary outcomes included 28-day mortality, in-hospital mortality, length of hospital stay, and length of ICU stay.

### 2.4. Statistical analysis

Continuous variables were determined for normality by the Kolmogorov–Smirnov test, those that conformed to normal distribution were expressed as mean ± standard deviation, and the t-test was used for intergroup comparison; those that did not conform to normal distribution were expressed as median (Q1–Q3), and the Wilcoxon rank-sum test was used for intergroup comparison. Categorical variables were expressed as sample size (percentage), and the chi-square test was used for intergroup comparison. Binomial Logistic regression was performed to identify independent risk factors for mortality, and variables with a *p-*value less than 0.1 in univariable regression analysis were included in multivariable regression analysis. Survival analysis of the two groups of patients was performed, Kaplan–Meier curves were drawn, and log-rank test was used to determine whether there was a statistical difference. The propensity score matching (PSM) analysis was performed to reduce bias based on the following variables: age, gender, CCI, SOFA score, SAPS II score, and GCS score (1:1 nearest neighbor matching, a caliper of 0.05, without replacement), and the propensity scores were calculated by a logistic regression model. All analyses were performed with the Medcalc software (version 19.6.1) and *p*-values <0.05 were considered statistically significant.

## 3. Results

### 3.1. Baseline characteristics

**Figure 1:**
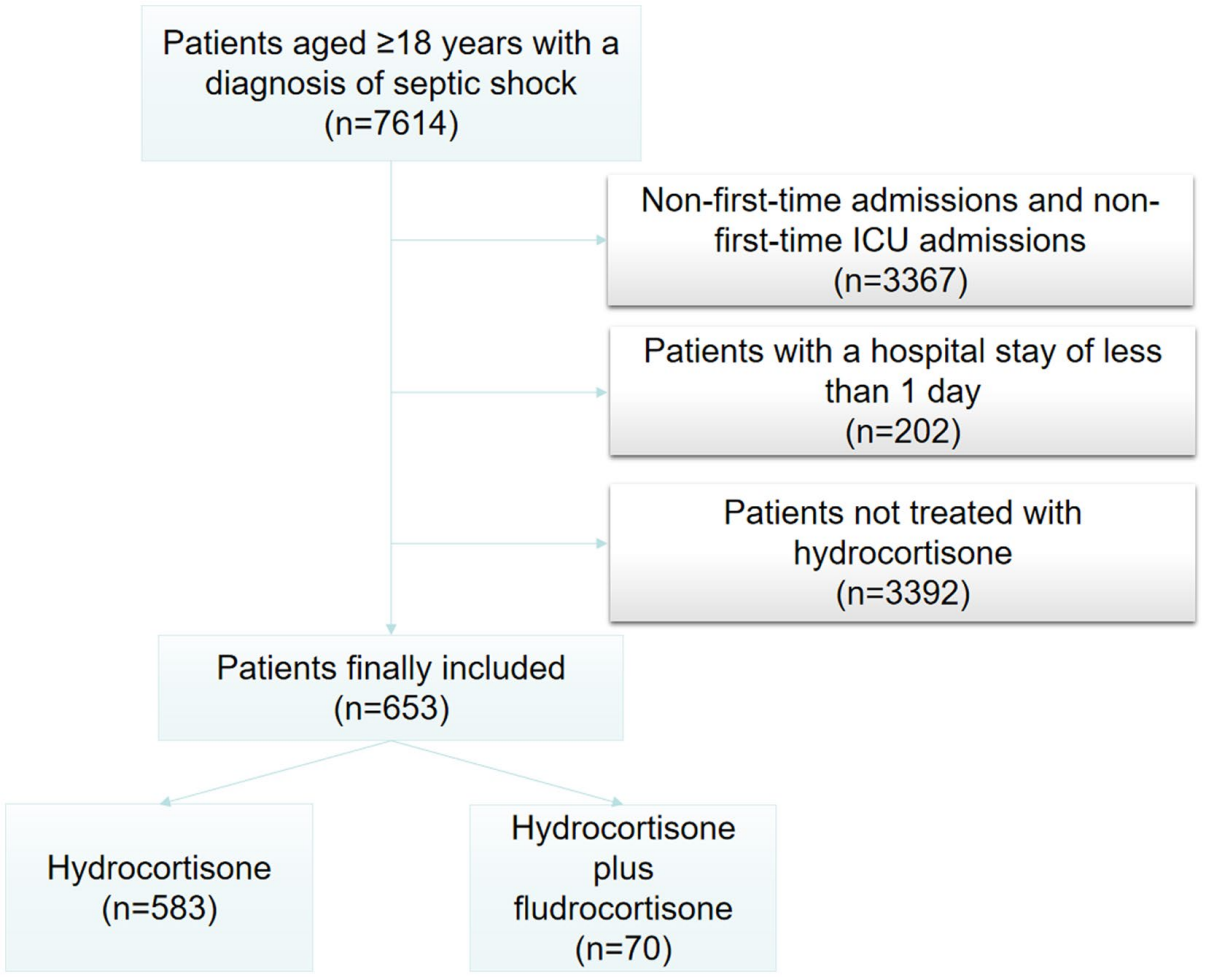
Flowchart of study cohort. ICU, intensive care unit.

A total of 653 patients were finally included in this study, of which 583 were treated with low-dose hydrocortisone alone, and 70 were treated with low-dose hydrocortisone plus fludrocortisone. The flow chart is shown in Figure 1. Table 1 presents the use of corticosteroids. In the hydrocortisone alone group, the dose of hydrocortisone was 300 mg (300–375 mg) per day, and the duration of hydrocortisone use was 2 days (3–5 days). In the hydrocortisone plus fludrocortisone group, the dose of hydrocortisone was 364 mg (300–400 mg) per day, and the duration of hydrocortisone use was 2 days (3–6 days); the dose of fludrocortisone was 0.1 mg (0.1–0.1 mg) per day, and the duration of fludrocortisone use was 2 days (3–6 days). For hydrocortisone, the vast majority of patients were prescribed 100 mg three times per day. There was no statistical difference in the dose and days of hydrocortisone use between the two groups. Supplementary Table S1 and Table 2 (after PSM) present the basic characteristics of the two groups of patients. The proportion of patients with acute kidney injury (AKI) and the proportion of RRT treatment in the hydrocortisone plus fludrocortisone group were higher than those in the hydrocortisone alone group, and there was no significant difference in other baseline characteristics.

**Table 1:** Details of glucocorticoid use.

	Hydrocortisone (*n* = 583)	Hydrocortisone plus fludrocortisone (*n* = 70)	*p*
Dosage (mg/day) of Hydrocortisone	300 (300–375)	364 (300–400)	0.07
Time (days) of hydrocortisone use	2 (3–5)	2 (3–6)	0.06
Dosage (mg/day) of Fludrocortisone	/	0.1 (0.1–0.1)	/
Time (days) of Fludrocortisone use	/	2 (3–6)	/

**Table 2:** Baseline characteristics of the study population (after PSM).

Characteristics	Hydrocortisone (*n* = 70)	Hydrocortisone plus fludrocortisone (*n* = 70)	*p*
*Age (year)	65.0 (57.0–77.0)	64.0 (56.0–72.0)	0.29
*Gender (Male)	34 (48.57)	40 (57.14)	0.31
Ethnicity			0.63
White	43 (61.43)	43 (61.43)	
Black	8 (11.42)	5 (7.14)	
Others	19 (27.14)	22 (31.43)	
*CCI	7 (5–9)	6 (4–9)	0.39
AKI	56 (80.00)	65 (92.86)	0.03
*SOFA score	12 (6–15)	13 (9–15)	0.36
*SAPS II score	51 (36–67)	50 (40–62)	0.94
*GCS score	13 (7–15)	13 (7–14)	0.66
Other therapies			
Epinephrine	63 (90.00)	66 (94.29)	0.35
Norepinephrine	63 (90.00)	66 (94.29)	0.35
Dopamine	12 (17.14)	10 (14.28)	0.64
Dobutamine	4 (5.71)	3 (4.29)	0.70
MV	38 (54.28)	47 (67.14)	0.12
ECMO	1 (1.42)	2 (2.86)	0.56
RRT	20 (28.57)	33 (47.14)	0.02

PSM, propensity score matching; CCI, Charlson comorbidity index; SOFA, sequential organ failure assessment; AKI, acute kidney injury; SAPS II, simplified acute physiology score ii; GCS, Glasgow Coma scale; MV, mechanical ventilation; ECMO, extracorporeal membrane oxygenation; RRT, renal replacement therapy. *Covariables included in the PSM.

### 3.2. Primary outcome

Among the 653 patients enrolled, 481 died at day 90 (mortality rate: 73.66%. The mortality rate of hydrocortisone alone group was 74.44%, while of hydrocortisone plus fludrocortisone group was 67.14%, *p* = 0.19). The relative risk (RR) of death was 1.11 (95% CI, 0.94–1.32) in favor of hydrocortisone plus fludrocortisone therapy (Supplementary Table S2). In multivariable regression analysis (Supplementary Table S3), CCI score and SAPS II score were independent risk factors for 90-day mortality (all *p*-values <0.05), and hydrocortisone plus fludrocortisone was not associated with 90-day mortality (OR = 0.70, 95%CI 0.41–1.20, *p* = 0.19). The median survival time was 32 days (95% CI 26–42 days) in the hydrocortisone alone group and 26 days (95% CI 16–174 days) in the hydrocortisone plus fludrocortisone group. Compared with the hydrocortisone alone group, the hazard ratio (HR) of the hydrocortisone plus fludrocortisone group was 1.00 (95%CI 0.74–1.35) (log-rank *p* = 0.99) (Supplementary Figure S1).

**Figure 2:**
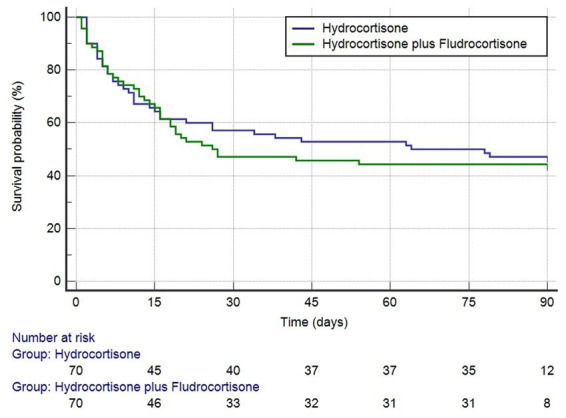
Kaplan–Meier survival curves (after PSM) for 90-days mortality based on hydrocortisone plus or without fludrocortisone (log-rank *p* = 0.2852).

After PSM, the RR of death was 1.07 (95% CI, 0.75–1.51) in favor of hydrocortisone plus fludrocortisone therapy (Table 3). As shown in Table 4, no independent risk factors for 90-day mortality were identified. The median survival time was 64 days (95% CI 16–289 days) in the hydrocortisone alone group and 26 days (95% CI 16–174 days) in the hydrocortisone plus fludrocortisone group. Compared with the hydrocortisone alone group, the HR of the hydrocortisone plus fludrocortisone group was 0.80 (95%CI 0.54–1.20) (log-rank *p* = 0.29) (Figure 2).

**Table 3:** Outcomes of the study population (after PSM).

Outcomes	Hydrocortisone (*n* = 70)	Hydrocortisone plus fludrocortisone (*n* = 70)	Relative risk* (95% CI)	*p*
Death at day 90	49 (70.00)	47 (67.14)	1.07 (0.75–1.51)	0.72
Death at day 28	30 (42.86)	37 (52.86)	0.82 (0.59–1.14)	0.24
Death in hospital	32 (45.71)	40 (57.14)	0.79 (0.57–1.11)	0.18
LOS hospital (day)	10.9 (4.8–20.8)	13.9 (6.6–23.8)	/	0.34
LOS ICU (day)	3.7 (2.0–10.0)	6.0 (2.4–11.7)	/	0.14

CI, confidence interval; LOS, length of stay; ICU, intensive care unit. *Shown is the relative risk for hydrocortisone plus fludrocortisone versus hydrocortisone alone.

**Table 4:** Binomial logistic regression analysis for 90-days mortality among patients with septic shock treated with hydrocortisone (after PSM).

	Univariable		Multivariable	
	OR (95% CI)	*p*	OR (95% CI)	*p*
CCI	1.19 (1.05–1.35)	<0.01	1.14 (0.99–1.30)	0.05
SAPS II	1.03 (1.01–1.05)	<0.01	1.02 (0.99–1.05)	0.07
Vasopressors	2.33 (0.64–8.52)	0.20		
MV	0.96 (0.46–2.00)	0.92		
ECMO	0.92 (0.08–10.36)	0.94		
RRT	0.83 (0.40–1.72)	0.61		
Plus Flud	0.88 (0.43–1.79)	0.72		

OR, Odds ratio; CI, confidence interval; CCI, Charlson comorbidity index; SAPS II, simplified acute physiology score II; MV, mechanical ventilation; ECMO, extracorporeal membrane oxygenation; RRT, renal replacement therapy; Flud, fludrocortisone. Vasopressors include epinephrine, norepinephrine, dopamine, and dobutamine.

### 3.3. Secondary outcomes

Among the 653 patients enrolled, 317 died at day 28 (mortality rate: 48.55%. The mortality rate of hydrocortisone alone group was 48.02%, while of hydrocortisone plus fludrocortisone group was 52.86%, *p* = 0.45). The RR of death was 0.91 (95% CI, 0.71–1.15)/0.82 (95% CI, 0.59–1.14) (after PSM) in favor of hydrocortisone plus fludrocortisone therapy (Supplementary Table S2 and Table 3). As shown in Supplementary Table S4, CCI score, SAPS II score, whether vasopressors was used, and whether RRT was used were independent risk factors for 28-day mortality (all *p*-values <0.05), and hydrocortisone plus fludrocortisone was not associated with 28-day mortality (OR = 1.21, 95%CI 0.74–2.00, *p* = 0.45). After PSM, only the SAPS II score (OR = 1.04, 95%CI 1.02–1.06, *p* < 0.01) was an independent risk factor for death (Supplementary Table S6). The median survival time could not be estimated in the hydrocortisone alone group due to too few deaths at 28 days. The median survival in the hydrocortisone plus fludrocortisone group was 26 days (95% CI 16–27 days). Compared with the hydrocortisone alone group, the HR of the hydrocortisone plus fludrocortisone group was 0.91 (95%CI 0.63–1.30, log-rank *p* = 0.60)/0.79 (95%CI 0.49–1.29, log-rank *p* = 0.35) (after PSM) (Supplementary Figures S2, S4).

Three hundred and forty-eight patients died in hospital, and the in-hospital mortality rate was 53.29% (the in-hospital mortality of hydrocortisone alone group was 52.83%, while of hydrocortisone plus fludrocortisone group was 57.14%, *p* = 0.50). The RR of death was 0.93 (95% CI, 0.74–1.15)/0.79 (95% CI, 0.57–1.11) (after PSM) in favor of hydrocortisone plus fludrocortisone therapy (Supplementary Table S2 and Table 3). As shown in Supplementary Table S5, CCI score, SAPS II score, whether vasopressors was used, and whether RRT was used were independent risk factors for in-hospital mortality (all *p*-values <0.05), and hydrocortisone plus fludrocortisone was not associated with in-hospital mortality (OR = 1.19, 95%CI 0.72–1.96, *p* = 0.50). After PSM, only the SAPS II score (OR = 1.04, 95%CI 1.01–1.06, *p* ≤ 0.01) was an independent risk factor for death (Supplementary Table S7). The median survival time was 23 days (95% CI 18–27 days) in the hydrocortisone alone group and 20 days (95% CI 16–28 days) in the hydrocortisone plus fludrocortisone group. Compared with the hydrocortisone alone group, the HR of the hydrocortisone plus fludrocortisone group was 0.98 (95%CI 0.70–1.35) (log-rank *p* = 0.88) (Supplementary Figure S3). After PSM, the median survival time was 33 days (95% CI 12–206 days) in the hydrocortisone alone group and 20 days (95% CI 16–28 days) in the hydrocortisone plus fludrocortisone group. Compared with the hydrocortisone alone group, the HR of the hydrocortisone plus fludrocortisone group was 0.90 (95%CI 0.56–1.43) (log-rank *p* = 0.65) (Supplementary Figure S5).

As presented in Supplementary Table S2, the median length of hospital stay was 11.6 days (5.4–23.1 days) in the hydrocortisone alone group and 13.9 days (6.6–23.8 days) in the hydrocortisone plus fludrocortisone group, and the difference was not statistically significant; the median length of ICU stay was 4.7 days (2.0–10.8 days) in the hydrocortisone alone group and 6.0 days (2.4–11.7 days) in the hydrocortisone plus fludrocortisone group, and the difference was still not statistically significant. After PSM, the median length of hospital stay was 10.9 days (4.8–20.8 days) and the median length of ICU stay was 3.7 days (2.0–10.0 days) in the hydrocortisone alone group, and the difference was still not statistically significant (Table 3).

## 4. Discussion

This study firstly provides a comprehensive report on the short-term mortality of patients with septic shock treated with hydrocortisone plus fludrocortisone versus hydrocortisone alone. Since the patients included in this study were all admitted to the ICUs and all had been started on low-dose hydrocortisone therapy, they were probably more severely ill than others. Therefore, our reported mortality rate appears to be higher than in previous studies (90-day mortality was 73.66%, 28-day mortality was 48.55%, and in-hospital mortality was 53.29%). Our study showed that co-administration of fludrocortisone did not reduce 90-day, 28-day, and in-hospital mortality in all patients with septic shock who started hydrocortisone therapy, nor did it affect the length of hospital stay and ICU stay. Our study also found that independent risk factors for short-term mortality (in-hospital mortality, 28-day mortality) included CCI, SAPS II, and two therapies (vasopressors and RRT), among which CCI can be used to assess comorbidity-related mortality risk (9), while higher SAPS II scores indicate more severe septic shock (8). However, with longer follow-up, 90-day mortality was independently associated with CCI and SAPS II, suggesting that mortality is closely related to comorbidities and disease severity when patients are treated as adequately as possible. However, after PSM, only SAPS II score was an independent risk factor for 28-day mortality and in-hospital mortality.

Corticosteroids reduce inflammation in various organs in septic animals and patients (10), which is the rationale for the use of corticosteroids in septic shock patients. Among the corticosteroid drugs in this study, hydrocortisone is a glucocorticoid, and fludrocortisone is a mineralocorticoid. Corticosteroids restore effective blood volume by increasing mineralocorticoid activity, and increase systemic vascular resistance, thereby improving cardiovascular function (11), which is the rationale for the treatment of septic shock with hydrocortisone plus fludrocortisone. As a result, the combination not only exerts a powerful anti-inflammatory effect through hydrocortisone, but also provides additional mineralocorticoid potency through fludrocortisone (hydrocortisone also synergistically increases the activity of fludrocortisone). However, to achieve this effect, the premise is that the mineralocorticoid receptor must be modulated by fludrocortisone, thereby mediating vasoconstriction to exert an anti-shock effect. If the patient’s organ failure has resulted in damage to the target of fludrocortisone, fludrocortisone is virtually incapable of exerting a physiological effect. In fact, the daily dose of 200 mg of hydrocortisone already has strong mineralocorticoid activity (12), perhaps adding fludrocortisone is unnecessary in this case, which may be the fundamental reason why hydrocortisone plus fludrocortisone has no survival benefit for the patients in this study. Moreover, improvements in cardiovascular function do not necessarily mean a reduction in mortality. After fluid resuscitation in septic shock, excessive volume overload in turn increases the burden on the heart and induces myocardial dysfunction. Meanwhile, unnecessarily high doses of inotropes may also exacerbate myocardial damage (13), thereby increasing the risk of death.

The prognosis of septic shock varies widely, with different studies reporting different responses to corticosteroids. On the one hand, there are differences in the design of individual studies, on the other hand, the current definition and severity stratification of septic shock is still not clear enough. The Third International Consensus Definitions for Sepsis and Septic Shock (Sepsis-3) further standardizes the definition of septic shock (1), but it is still not completely accurate to describe it from a pathological and physiological perspective. For example, according to the Delphi consensus process, elevated lactate levels are also an important feature of septic shock (1); while Ait-Oufella et al. (14) found that delayed capillary refill, as one of the criteria for tissue hypoperfusion, has profound implications for the diagnosis of septic shock. These potential diagnostic criteria facilitate accurate identification of septic shock, but also present a challenge for the use of corticosteroids for septic shock. Circulatory dysfunction in septic shock also varies widely, and it has been suggested that the severity of septic shock can be classified according to the dose of catecholamine administered and the measurement of serum lactate levels, and stratified according to the dose of catecholamine, may help determine which patients would benefit from corticosteroid use (15). On the whole, for the corticosteroid therapy of septic shock, it may be necessary to implement individualized and precise treatment, that is, to select the appropriate therapy for different primary diseases, different types and different severities of septic shock.

The strengths of this study lie in its relatively large sample size and reliable data quality. The baseline characteristics of the two groups of patients in this study were basically balanced and comparable, thus our findings are convincing. However, this study has certain limitations. Firstly, we cannot assess the adverse events directly caused by corticosteroids, and we cannot determine whether death due to adverse events offsets the survival benefit provided by corticosteroids; secondly, due to the limitations of the MIMIC database, this study did not quantify other outcomes, including the number of days that patients were alive and free of vasopressors, the time to weaning from mechanical ventilation, the number of days free of organ failure, and whether shock state of the patient has been reversed due to drug use, etc.; in addition, the doses/days of use of the two corticosteroids in this study were slightly different from previous studies. In a systematic review, only 2 of 33 trials were sufficiently powered to elucidate the effect of long-term (≥5 days) low-dose corticosteroid treatment on mortality (16), but in our view, the timing of dosing may be one of the potential reasons why the addition of fludrocortisone did not help reduce mortality (aside from the normality of treatment time, the patients in this study were treated with hydrocortisone for a mean of 4.7 days and fludrocortisone for a mean of 4.5 days); finally, since there is currently no test that can reliably diagnose adrenal insufficiency in critically ill patients (17), we also cannot determine adrenal function in patients (8) and determine whether any patients would reasonably benefit from corticosteroid therapy. In a word, we found that co-administration of fludrocortisone did not confer a survival benefit in septic shock patients treated with hydrocortisone, but given the limitations of retrospective studies and the MIMIC database, we advocate conducting prospective clinical randomized controlled trials to further confirm this point of view.

## 5. Conclusion

In the treatment of patients with septic shock, hydrocortisone plus fludrocortisone did not reduce 90-day mortality, 28-day mortality, and in-hospital mortality compared with hydrocortisone alone, and had no effect on the length of hospital stay and ICU stay. Due to the limitations of retrospective study design and MIMIC-IV database, the conclusions of this study still need to be confirmed by prospective clinical randomized controlled trials.

## Data availability statement

Publicly available datasets were analyzed in this study. This data can be found here: https://mimic.mit.edu/.

## Ethics statement

This study was ethically approved by an affiliate of the Massachusetts Institute of Technology (No. 27653720). Written informed consent for participation was not required for this study in accordance with the national legislation and the institutional requirements.

## Author contributions

XC and ZF: conceptualization, formal analysis, investigation, software, methodology, resources, visualization, and writing – original draft. YL and XZ: formal analysis, investigation, and software. TH: project administration, supervision validation, and writing-review and editing. All authors have approved the final version of the manuscript for submission and agree to be accountable for all aspects of the work.

## Conflict of interest

The authors declare that the research was conducted in the absence of any commercial or financial relationships that could be construed as a potential conflict of interest.

## Publisher’s note

All claims expressed in this article are solely those of the authors and do not necessarily represent those of their affiliated organizations, or those of the publisher, the editors and the reviewers. Any product that may be evaluated in this article, or claim that may be made by its manufacturer, is not guaranteed or endorsed by the publisher.

## Footnotes

^1^https://mimic.mit.edu/

## Abbreviations

ICU: intensive care unit
CCI: Charlson comorbidity index
SAPS II: Simplified Acute Physiology Score II
CORTICUS: the Corticosteroid Therapy of Septic Shock trial
APROCCHSS: the Activated Protein C and Corticosteroids for Human Septic Shock trial
MIMIC: Medical Information Mart for Intensive Care
NIH: National Institutes of Health
SQL: structure query language
SOFA: Sequential Organ Failure Assessment
GCS: Glasgow Coma Scale
ECMO: extracorporeal membrane oxygenation
RRT: renal replacement therapy
AKI: acute kidney injury
HR: hazard ratio
PSM: propensity score matching
