# Supplementary material for: Association of mortality with fludrocortisone addition to hydrocortisone treatment among septic shock patients: a propensity score matching analysis
Source: Front Med (Lausanne). 2023 May 9;10:1190758. doi: 10.3389/fmed.2023.1190758 (PMC10203469; doi:10.3389/fmed.2023.1190758)
Supplement: Supplementary file 1 [file Data_Sheet_1.doc]

**Supplementary Materials**

**Table S1 Baseline characteristics of the study population**

| **Characteristics** | **Hydrocortisone(n=583)** | **Hydrocortisone plus Fludrocortisone (n=70)** | ***p*** |
| --- | --- | --- | --- |
| **Age (year)** | 67.0 (57.0-76.5) | 64.0 (56.0-72.0) | 0.12 |
| **Gender (Male)** | 300 (51.45) | 40 (57.14) | 0.37 |
| **Ethnicity** |  |  | 0.32 |
| White | 397 (68.10) | 43 (61.43) |  |
| Black | 50 (8.58) | 5 (7.14) |  |
| Others | 136 (23.32) | 22 (31.43) |  |
| **CCI** | 6 (5-9) | 6 (4-9) | 0.92 |
| **AKI** | 487 (83.53) | 65 (92.86) | 0.04 |
| **SOFA score** | 11 (7-15) | 13 (9-15) | 0.07 |
| **SAPS II score** | 51 (40-65) | 50 (40-62) | 0.88 |
| **GCS score** | 13 (6-14) | 13 (7-14) | 0.90 |
| **Other therapies** |  |  |  |
| Epinephrine | 533 (91.42) | 66 (94.29) | 0.41 |
| Norepinephrine | 531 (91.08) | 66 (94.29) | 0.37 |
| Dopamine | 56 (9.61) | 10 (14.28) | 0.22 |
| Dobutamine | 38 (6.52) | 3 (4.29) | 0.47 |
| MV | 342 (58.66) | 47 (67.14) | 0.17 |
| ECMO | 6 (1.01) | 2 (2.86) | 0.21 |
| RRT | 186 (31.90) | 33 (47.14) | 0.01 |

CCI=Charlson Comorbidity Index, SOFA=Sequential Organ Failure Assessment, AKI=Acute Kidney Injury, SAPS II=Simplified Acute Physiology Score II, GCS=Glasgow Coma Scale, MV=Mechanical Ventilation, ECMO=Extracorporeal Membrane Oxygenation, RRT=Renal Replacement Therapy.

**Table S2 Outcomes of the study population**

| **Outcomes** | **Hydrocortisone(n=583)** | **Hydrocortisone plus Fludrocortisone (n=70)** | **Relative Risk***  **(95% CI)** | ***p*** |
| --- | --- | --- | --- | --- |
| Death at day 90 | 434 (74.44) | 47 (67.14) | 1.11 (0.94-1.32) | 0.19 |
| Death at day 28 | 280 (48.02) | 37 (52.86) | 0.91 (0.72-1.15) | 0.45 |
| Death in hospital | 308 (52.83) | 40 (57.14) | 0.93 (0.74-1.15) | 0.49 |
| LOS hospital (day) | 11.6 (5.4-23.1) | 13.9 (6.6-23.8) | / | 0.28 |
| LOS ICU (day) | 4.7 (2.0-10.8) | 6.0 (2.4-11.7) | / | 0.37 |

CI=Confidence Interval, LOS= Length of Stay, ICU= Intensive Care Unit.

*Shown is the relative risk for hydrocortisone plus fludrocortisone versus hydrocortisone alone.

**Table S3 Binomial Logistic regression analysis for 90-days mortality among patients with septic shock treated with hydrocortisone**

|  | **Univariable** | |  | **Multivariable** | |
| --- | --- | --- | --- | --- | --- |
|  | **OR（95% CI）** | ***p*** |  | **OR（95% CI）** | ***p*** |
| **CCI** | 1.29(1.20-1.38) | ＜0.01 |  | 1.24(1.15-1.34) | ＜0.01 |
| **SAPS II** | 1.04(1.02-1.05) | ＜0.01 |  | 1.02(1.01-1.03) | ＜0.01 |
| **Vasopressors** | 2.13(1.20-3.79) | 0.01 |  | 1.76 (0.93-3.34) | 0.08 |
| **MV** | 1.12(0.79-1.59) | 0.53 |  |  |  |
| **ECMO** | 1.07(0.22-5.37) | 0.93 |  |  |  |
| **RRT** | 1.43(0.97-2.09) | 0.07 |  | 1.13(0.73-1.73) | 0.59 |
| **Plus Flud** | 0.70(0.41-1.20) | 0.19 |  |  |  |

OR=Odds Ratio, CI=Confidence Interval, CCI=Charlson Comorbidity Index, SAPS II=Simplified Acute Physiology Score II, MV=Mechanical Ventilation, ECMO=Extracorporeal Membrane Oxygenation, RRT=Renal Replacement Therapy, Flud=Fludrocortisone.

Vasopressors include epinephrine, norepinephrine, dopamine and dobutamine.

**Table S4 Binomial Logistic regression analysis for 28-days mortality among patients with septic shock treated with hydrocortisone**

|  | **Univariable** | |  | **Multivariable** | |
| --- | --- | --- | --- | --- | --- |
|  | **OR（95% CI）** | ***p*** |  | **OR（95% CI）** | ***p*** |
| **CCI** | 1.17 (1.11-1.24) | ＜0.01 |  | 1.12(1.05-1.19) | ＜0.01 |
| **SAPS II** | 1.04(1.03-1.05) | ＜0.01 |  | 1.03(1.02-1.04) | ＜0.01 |
| **Vasopressors** | 2.84(1.51-5.34) | ＜0.01 |  | 2.03(1.03-4.01) | 0.04 |
| **MV** | 1.59(1.16-2.18) | ＜0.01 |  | 0.87(0.59-1.29) | 0.50 |
| **ECMO** | 1.06(0.26-4.28) | 0.93 |  |  |  |
| **RRT** | 1.99(1.43-2.76) | ＜0.01 |  | 1.52(1.06-2.19) | 0.03 |
| **Plus Flud** | 1.21(0.74-1.99) | 0.45 |  |  |  |

OR=Odds Ratio, CI=Confidence Interval, CCI=Charlson Comorbidity Index, SAPS II=Simplified Acute Physiology Score II, MV=Mechanical Ventilation, ECMO=Extracorporeal Membrane Oxygenation, RRT=Renal Replacement Therapy, Flud=Fludrocortisone.

Vasopressors include epinephrine, norepinephrine, dopamine and dobutamine.

**Table S5 Binomial Logistic regression analysis for in-hospital mortality among patients with septic shock treated with hydrocortisone**

|  | **Univariable** | |  | **Multivariable** | |
| --- | --- | --- | --- | --- | --- |
|  | **OR（95% CI）** | ***p*** |  | **OR（95% CI）** | ***p*** |
| **CCI** | 1.17(1.10-1.23) | ＜0.01 |  | 1.11(1.04-1.18) | ＜0.01 |
| **SAPS II** | 1.04(1.03-1.05) | ＜0.01 |  | 1.03(1.02-1.04) | ＜0.01 |
| **Vasopressors** | 3.50(1.86-6.58) | ＜0.01 |  | 2.66(1.35-5.26) | ＜0.01 |
| **MV** | 1.47(1.06-1.99) | 0.02 |  | 0.72(0.49-1.08) | 0.11 |
| **ECMO** | 2.66(0.53-13.26) | 0.23 |  |  |  |
| **RRT** | 2.23(1.59-3.13) | ＜0.01 |  | 1.74(1.20-2.53) | ＜0.01 |
| **Plus Flud** | 1.19(0.72-1.96) | 0.50 |  |  |  |

OR=Odds Ratio, CI=Confidence Interval, CCI=Charlson Comorbidity Index, SAPS II=Simplified Acute Physiology Score II, MV=Mechanical Ventilation, ECMO=Extracorporeal Membrane Oxygenation, RRT=Renal Replacement Therapy, Flud=Fludrocortisone.

Vasopressors include epinephrine, norepinephrine, dopamine and dobutamine.

**Table S6 Binomial Logistic regression analysis for 28-days mortality among patients with septic shock treated with hydrocortisone (after PSM)**

|  | **Univariable** | |  | **Multivariable** | |
| --- | --- | --- | --- | --- | --- |
|  | **OR（95% CI）** | ***p*** |  | **OR（95% CI）** | ***p*** |
| **CCI** | 1.12(1.0-1.25) | 0.04 |  | 1.04(0.93-1.17) | 0.49 |
| **SAPS II** | 1.04(1.02-1.06) | ＜0.01 |  | 1.04(1.02-1.06) | ＜0.01 |
| **Vasopressors** | 1.41(0.38-5.23) | 0.61 |  |  |  |
| **MV** | 1.49(0.75-2.96) | 0.25 |  |  |  |
| **ECMO** | 2.22(0.20-25.01) | 0.52 |  |  |  |
| **RRT** | 1.22(0.62-2.42) | 0.57 |  |  |  |
| **Plus Flud** | 1.50(0.77-2.91) | 0.24 |  |  |  |

OR=Odds Ratio, CI=Confidence Interval, CCI=Charlson Comorbidity Index, SAPS II=Simplified Acute Physiology Score II, MV=Mechanical Ventilation, ECMO=Extracorporeal Membrane Oxygenation, RRT=Renal Replacement Therapy, Flud=Fludrocortisone.

Vasopressors include epinephrine, norepinephrine, dopamine and dobutamine.

**Table S7 Binomial Logistic regression analysis for in-hospital mortality among patients with septic shock treated with hydrocortisone (after PSM)**

|  | **Univariable** | |  | **Multivariable** | |
| --- | --- | --- | --- | --- | --- |
|  | **OR（95% CI）** | ***p*** |  | **OR（95% CI）** | ***p*** |
| **CCI** | 1.13(1.01-1.26) | 0.03 |  | 1.06(0.94-1.19) | 0.34 |
| **SAPS II** | 1.04(1.02-1.06) | ＜0.01 |  | 1.04(1.01-1.06) | ＜0.01 |
| **Vasopressors** | 2.64(0.65-10.66) | 0.17 |  |  |  |
| **MV** | 1.32(0.67-2.60) | 0.43 |  |  |  |
| **ECMO** | 1.91(0.17-21.61) | 0.60 |  |  |  |
| **RRT** | 1.24(0.62-2.45) | 0.54 |  |  |  |
| **Plus Flud** | 1.58(0.81-3.09) | 0.18 |  |  |  |

OR=Odds Ratio, CI=Confidence Interval, CCI=Charlson Comorbidity Index, SAPS II=Simplified Acute Physiology Score II, MV=Mechanical Ventilation, ECMO=Extracorporeal Membrane Oxygenation, RRT=Renal Replacement Therapy, Flud=Fludrocortisone.

Vasopressors include epinephrine, norepinephrine, dopamine and dobutamine.


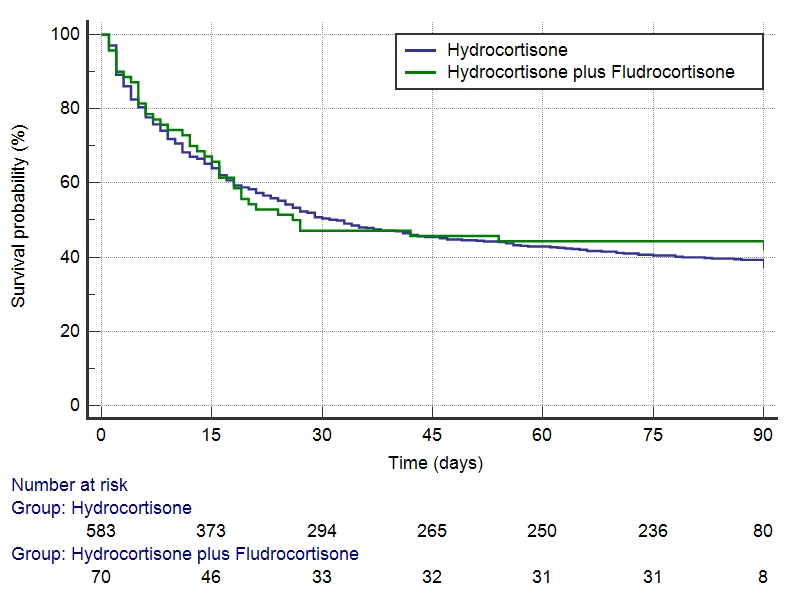


**Fig. S1 Kaplan-Meier survival curves for 90-days mortality based on hydrocortisone plus or without fludrocortisone (log-rank *p* =0.99).**


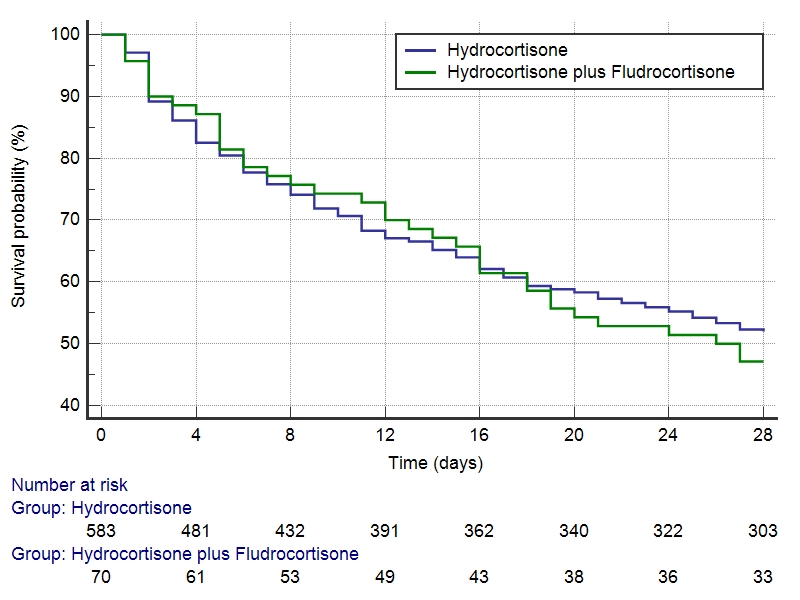


**Fig. S2 Kaplan-Meier survival curves for 28-days mortality based on hydrocortisone plus or without fludrocortisone (log-rank *p* =0.60).**


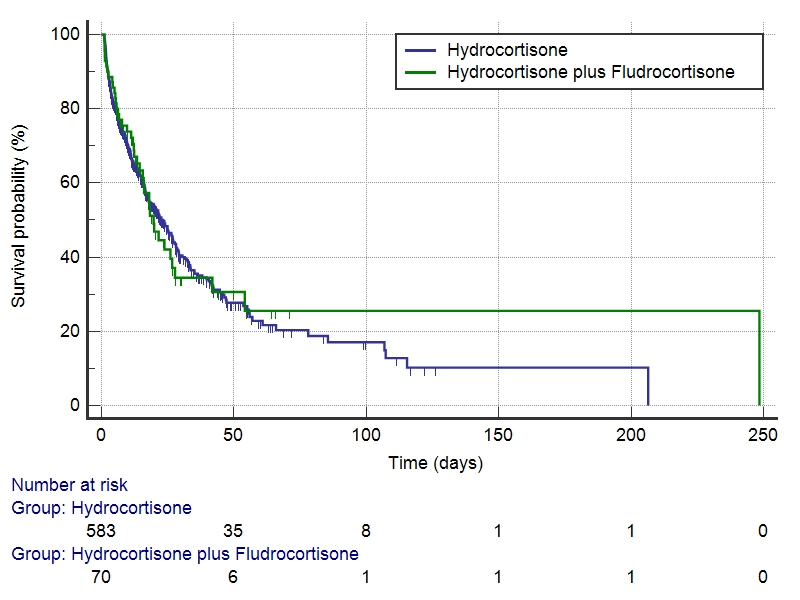


**Fig. S3 Kaplan-Meier survival curves for in-hospital mortality based on hydrocortisone plus or without fludrocortisone (log-rank *p* =0.88).**


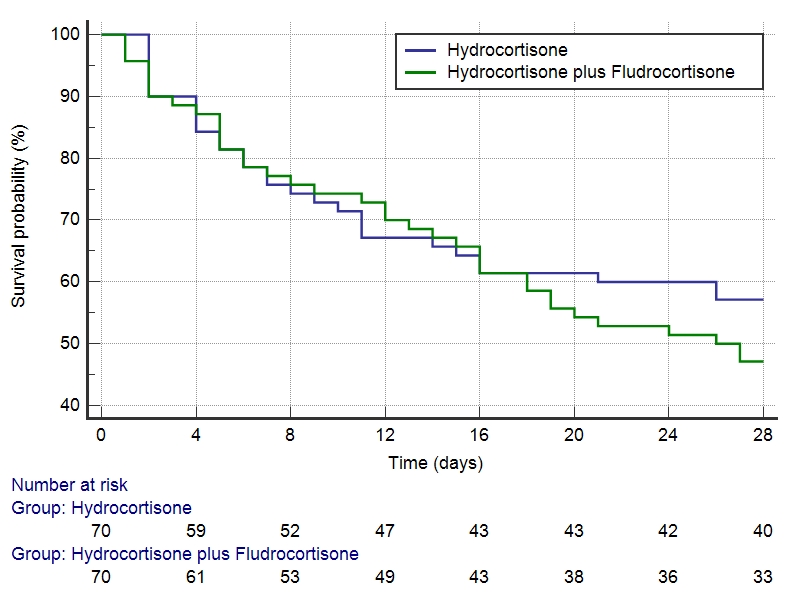


**Fig. S4 Kaplan-Meier survival curves (after PSM) for 28-days mortality based on hydrocortisone plus or without fludrocortisone (log-rank *p* =0.35).**


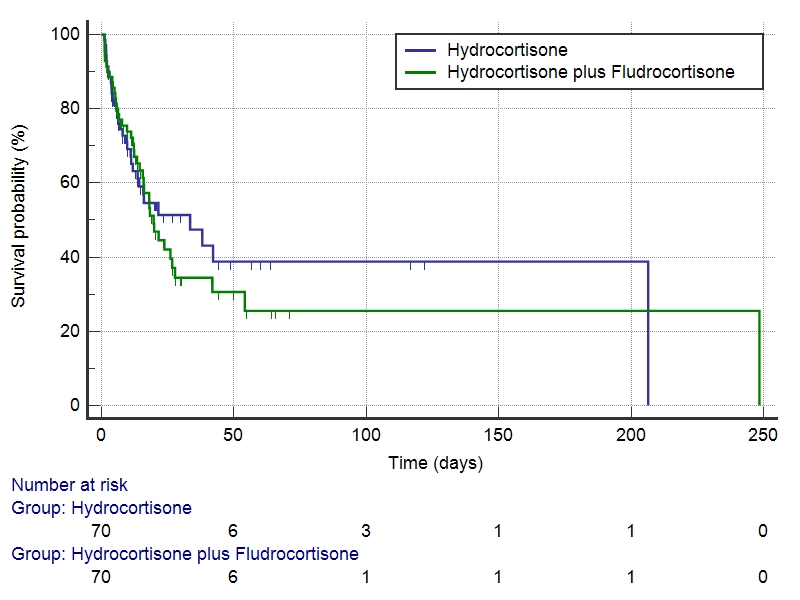


**Fig. S5 Kaplan-Meier survival curves (after PSM) for in-hospital mortality based on hydrocortisone plus or without fludrocortisone (log-rank *p* =0.65).**
